# Genome-wide analysis of the acyl-coenzyme A synthetase family and their association with the formation of goat milk flavour

**DOI:** 10.3389/fgene.2022.980463

**Published:** 2022-09-07

**Authors:** Fuhong Zhang, Jun Luo, Chenbo Shi, Lu Zhu, Qiuya He, Huibin Tian, Jiao Wu, Jianqing Zhao, Cong Li

**Affiliations:** Key Laboratory of Animal Genetics, Breeding and Reproduction of Shaanxi Province, College of Animal Science and Technology, Northwest A&F University, Yangling, China

**Keywords:** acyl-coenzyme A synthetase (ACSs), genome-wide, phylogenetic analysis, dairy goat, goaty flavour

## Abstract

Goat milk is rich in fat and protein, thus, has high nutritional values and benefits human health. However, goaty flavour is a major concern that interferes with consumer acceptability of goat milk and the 4-alkyl-branched-chain fatty acids (vBCFAs) are the major substances relevant to the goaty flavour in goat milk. Previous research reported that the acyl-coenzyme A synthetases (ACSs) play a key role in the activation of fatty acids, which is a prerequisite for fatty acids entering anabolic and catabolic processes and highly involved in the regulation of vBCFAs metabolism. Although ACS genes have been identified in humans and mice, they have not been systematically characterized in goats. In this research, we performed genome-wide characterization of the ACS genes in goats, identifying that a total of 25 ACS genes (without *ACSM2A*) were obtained in the *Capra hircus* and each ACS protein contained the conserved AMP-binding domain. Phylogenetic analysis showed that out of the 25 genes, 21 belonged to the ACSS, ACSM, ACSL, ACSVL, and ACSBG subfamilies. However, *AACS*, *AASDH*, *ACSF*, and *ACSF3* genes were not classified in the common evolutionary branch and belonged to the ACS superfamily. The genes in the same clade had similar conserved structures, motifs and protein domains. The expression analysis showed that the majority of ACS genes were expressed in multi tissues. The comparative analysis of expression patterns in non-lactation and lactation mammary glands of goat, sheep and cow indicated that *ACSS2* and *ACSF3* genes may participate in the formation mechanisms of goaty flavour in goat milk. In conclusion, current research provides important genomic resources and expression information for ACSs in goats, which will support further research on investigating the formation mechanisms of the goaty flavour in goat milk.

## Introduction

The ACS family comprises a large and diverse group of enzymes. Each member of the ACS family contains a highly conserved amino acid sequence motif, an ATP/AMP binding domain (Black et al., 1997; Mashek et al., 2004). This motif locates at 200–300 amino acids from the N-terminus and is the marker of adenylate-forming enzymes (Watkins et al., 2007; Watkins and Ellis, 2012). In humans, the ACS gene family contains 26 members. Of these members, 22 are subdivided into five subfamilies based on their discrepancies in AMP/ATP and fatty acid-binding motifs (Steinberg et al., 2000). The five subfamilies are the short-chain acyl-CoA synthetase (ACSS), the medium-chain acyl-CoA synthetase (ACSM), the long-chain acyl-CoA synthase (ACSL), the very long-chain synthetase (ACSVL) and the bubblegum ACS synthetase (ACSBG) subfamilies (Grevengoed et al., 2014). Due to their structural features, the acetoacetyl-CoA synthetase (*AACS*), acyl-CoA synthetase family member 2 (*ACSF2*), acyl-CoA synthetase family member 3 (*ACSF3*), and 2-Aminoadipic 6-semialdehyde dehydrogenase (*AASDH*) genes are not classified into any subfamilies and are independent members of the ACS superfamily (Watkins et al., 2007). HUGO nomenclature advisors suggested name these four genes using the interim designation ACSF (ACSF1–4) family (Watkins et al., 2007). In mammals, the ACS family has been characterized in humans and mice, but not in goats.

Different ACS subfamilies exhibit their own preferences for different length of fatty acids (Rossi Sebastiano and Konstantinidou, 2019), with members of each subfamily showing tissue different expression profiles and subcellular locations (Grevengoed et al., 2014). Fatty acids with less than 6 carbons are typically catalyzed by ACSSs, C6—C10 fatty acids are catalyzed by ACSMs, while C12—C20 fatty acids and very long chain fatty acids (>20 carbons) are preferred by ACSLs and ACSVLs, respectively (Grevengoed et al., 2014; Tang et al., 2018). The long chain fatty acids are predominant fats and fulfill essential physiological functions in living organisms (O'Brien et al., 2020). ACSL family has been widely researched in the past (Ansari et al., 2017). ACSL family members, including *ACSL1*, *ACSL3*, *ACSL4*, *ACSL5*, and *ACSL6*, exhibit distinct substrate preferences (Rossi Sebastiano and Konstantinidou, 2019). *ACSL1*, highly expressed in multi tissues such as liver, kidney heart and muscle (Grevengoed et al., 2014), typically prefers oleate and linoleate (Kanter et al., 2012). The absence of *ACSL1* inhibits the sensitivity of macrophages to oleic- and linoleic-mediated degradation of ABCA1 (ATP binding cassette transporter A1), and increase cholesterol spillage (Kanter et al., 2012). *ACSL3* has been found in the endoplasmic reticulum and lipid droplets (Grevengoed et al., 2014), which preferentially catalyzes palmitic and arachidonic fatty acids (Ndiaye et al., 2020). *ACSL4* has also prominent expressed in multi tissues, mainly adrenal gland, brain, ovary, and testis (Grevengoed et al., 2014), and prefers arachidonic acid (Ndiaye et al., 2020). Moreover, previous research suggests the dysregulated expression of both *ACSL3* and *ACSL4* is linked to several diseases, especially cancer (Tang et al., 2018). *ACSL5* has the highest expression in intestinal mucosa relative to other tissues (Meller et al., 2013). The splice variants in *ACSL5* is associated with several types of cancer (Perez-Nunez et al., 2019), and a deletion of *ACSL5* can lead to intestinal lipid malabsorption (O'Brien et al., 2020). The expression of *ACSL6* is large in brain (Takahiro and Tokuo, 1992; Grevengoed et al., 2014), and the absence of *ACLS6* is the most likely cause of the omega-3 docosahexaenoic acid (DHA) deficiency in the brain and spine (Fernandez et al., 2018). Taken altogether, ACSs fulfil distinct roles in fatty acid metabolism.

Goat milk is rich in milk fat and protein, which has high nutritional value and is beneficial to human health (Teng et al., 2018). However, the consumer’s acceptance of goat milk and dairy products is restricted because of the perceived characteristic goaty flavour (Kaffarnik et al., 2014). Previous studies have confirmed that 4-alkyl-branched-chain fatty acids (vBCFAs) are the main substances relevant to the goaty flavour in goat milk (Brennand et al., 2010; Salles et al., 2010; Teng et al., 2018). In addition, the concentration of vBCFAs, including 4-ethyloctanoic acid (4-Et-8:0), 4-methyloctanoic acid (4-Me-8:0) and 4-methylnonanoic acid (4-Me-9:0) in goat milk is much higher than in cow milk (Ha and Lindsay, 1993; Kaffarnik et al., 2014). Branched-chain fatty acids (BCFAs) are more likely to be synthesized in goat tissues rather than by rumen microbes (Berthelot et al., 2001). Fatty acids, including vBCFAs, are catalyzed to fatty acyl-CoAs by ACSs before involving in both anabolic and catabolic processes (Steinberg et al., 2000; Watkins et al., 2007; Grevengoed et al., 2014). However, research on the potential role of ACSs in the regulation of vBCFAs metabolism was limited.

Thus, the present study performed a genome-wide characterization of the ACS genes in goat and analyzed their expression profiles in dairy goat, and compared their expression patterns in the non-lactation and lactation mammary glands of dairy goat, sheep, and cow. The major objective of this study was to investigate the function of ACSs in the formation of goaty flavour in goat milk, thus, to provide gene resources for the genetic improvement of goaty flavour by regulating the ACS-mediated vBCFAs metabolism.

## Materials and methods

### Genome-wide identification of acyl-coenzyme A synthetase genes

The goat (*Capra hircus*) reference genome (Accession NO. GCA_001704415.1) was downloaded from the Ensemble database (http://ftp.ensembl.org/pub/release-104/fasta/capra_hircus/dna). The ACS protein sequences of *Homo sapiens* were downloaded from the GenBank (https://www.ncbi.nlm.nih.gov/). To identify ACS genes of goat, we used human ACS protein sequences as queries to carry out local BLASTP (*p* = 0.001) searches against the goat genome database. To further improve the accuracy, we acquired the HMM (hidden Markov model) profile of the AMP-binding domain (PF00501.31) from the Pfam database (http://pfam.xfam.org) (Kochan et al., 2009), then searched the sequence of candidate genes identified by blastp, using HMMER 3.3 (http://eddylab.org/software/hmmer/hmmer-3.3.tar.gz) (Finn et al., 2011). Using the same criterion, ACS genes protein sequences of sheep and cow were obtained from *Ovis aries* (Accession NO, GCA_002742125.1) and *Bos taurus* reference genomes (Accession NO. GCA_002263795.2).

Each potential ACS genes of goat was further analyzed by the programs Pfam to identify the location of domains. The “Compute pI/Mw” tool was used to obtain the pI (theoretical isoelectric point) and MW (molecular weight) of identified ACS proteins (https://web.expasy.org/compute_pi/). Subcellular localizations of identified ACS genes were predicted, using WoLF PSORT website (https://wolfpsort.hgc.jp/). The location of ACS genes on goat genome was mapped using an online website (http://mg2c.iask.in/mg2c_v2.1/). Motif prediction of identified ACS proteins was analyzed using the MEME suite with a maximum number of 15 motifs (http://meme-suite.org/tools/meme). The gene structures and motif sequences were drawn using the EvolView online tool (https://www.evolgenius.info/evolview/#login) (Balakrishnan et al., 2019).

### Multiple sequence alignment and phylogenetic tree construction

The ACS protein sequences from goat, human, sheep, and cow were analyzed together. Multiple sequence alignment was conducted using the MAFFT-7.429 (Katoh and Standley, 2013). The results of multiple sequence alignments were used to predict conserved domains and motifs, and to construct phylogenetic trees. The phylogenetic tree was constructed using the IQ-TREE with a ultrafast bootstrap value of 1,000 (Lam-Tung et al., 2015). The phylogenetic tree and locations of conserved domains of ACSs were drawn using the EvolView online tool.

### Expression analysis of acyl-coenzyme A synthetase genes

The expression levels of the ACS genes of dairy goat at non-lactation and lactation stages were analyzed based on the transcriptome data that were obtained by our laboratory (NCBI SRA accession: PRJNA637690) (Li et al., 2020). To understand the expression profiles of distinct tissues in dairy goat, transcriptome datasets of three organs (heart, kidney, and liver) and skeletal muscle tissue were downloaded, with 3 biological replicates (NCBI SRA accession: PRJNA309284 and NCBI SRA accession: PRJNA309345) from SRA database (https://www.ncbi.nlm.nih.gov/sra/). To further investigate the distinction in transcriptional responses of ACSs in the mammary glands of goat, sheep and cow, the sheep (NCBI SRA accession: PRJNA309284) and cow (NCBI SRA accession: PRJNA482783) transcriptome datasets of mammary gland in the non-lactation and lactation period were downloaded from SRA database (https://www.ncbi.nlm.nih.gov/sra/). The analysis procedures were described as bellow. The transcriptome datasets were converted to fastq format using fastq-dump. All clean reads were mapped to the respective reference genome sequence using Hisat2-2.1.0 (https://github.com/infphilo/hisat2/), and the transcription-level expression was calculated using StringTie (https://ccb.jhu.edu/software/stringtie/). The FPKM values were log_2_ transformed, and the heat map of gene expression levels was plotted using the EvolView online tool.

## Results

### Characterization of acyl-coenzyme A synthetase genes

To identify ACS genes in goat, ACS protein sequences of human were used as queries to search the goat genome. A total of 25 ACS genes (*ChACSs*) were identified after an analysis of conserved domains in goat. The full-length ChACS protein sequences were given in the Supplementary Sheet S1. We found that the *ACSM2A* gene was absent in goat reference genome, nor in sheep and cows’ reference genome. The *ChACSs* shared homology to human ACSs (*HsACSs*), with the amino acid identity of 71.2%–96.4% (Supplementary Sheet S2). Among 25 identified genes, 22 were divided into five groups based on the sequence similarity and the principle of the human ACS family nomenclature. There were three genes in ACSS subfamily, five genes in the ACSM subfamily, five genes in the ACSL subfamily, six genes in the ACSVL subfamily, and two genes in the ACSBG subfamily (Table 1). However, *ChAACS*, *ChACSF2*, *ChACSF3*, and *ChAASDH* were not classified in any subfamilies and they were independent members of the ACS superfamily (Table 2).

**TABLE 1:** Information of ACS family genes in goat.

Subfamily	Gene	CDS (bp)	Exon	Intron	Amino acid (aa)	MW (Da)	pI	Localization
ACSBG	*ChACSBG1*	2,298	17	16	766	85,738.59	6.54	Mitochondrion
	*ChACSBG2*	1866	14	13	622	69,142.06	7.87	Plasma membrane
ACSL	*ChACSL*1	2,100	21	20	700	78,128.85	8.00	Cytoplasm
	*ChACSL3*	2,163	15	14	721	80,127.67	8.65	Cytoplasm
	*ChACSL4*	2013	16	15	671	74,477.15	8.41	Mitochondrion
	*ChACSL5*	2052	21	20	684	75,532.52	7.50	Endoplasmic reticulum
	*ChACSL6*	2,169	21	20	723	80,846.28	6.14	Endoplasmic reticulum
ACSM	*ChACSM1*	1734	14	13	578	64,819.43	7.84	Mitochondrion
	*ChACSM2B*	1734	15	14	578	64,636.62	7.21	Mitochondrion
	*ChACSM3*	1743	14	13	581	65,712.44	8.86	Mitochondrion
	*ChACSM4*	1743	13	12	581	64,965.59	8.64	Mitochondrion
	*ChACSM5*	1773	14	13	591	66,162.68	7.56	Mitochondrion
ACSS	*ChACSS1*	2028	14	13	676	74,352.33	6.59	Mitochondrion
	*ChACSS2*	2,145	19	18	715	80,245.85	6.34	Cytoplasm
	*ChACSS3*	2061	16	15	687	74,761.3	8.86	Mitochondrion
ACSVL	*ChSLC27A1*	1941	13	12	647	71,003.95	8.83	Peroxisome
	*ChSLC27A2*	1863	10	9	621	70,319.22	8.72	Endoplasmic reticulum
	*ChSLC27A3*	2,211	11	10	737	79,697.36	8.73	Plasma membrane
	*ChSLC27A4*	1932	13	12	644	72,242.34	8.92	Peroxisome
	*ChSLC27A5*	2073	10	9	691	75,683.17	8.70	Plasma membrane
	*ChSLC27A6*	1905	10	9	635	71,710.21	8.71	Endoplasmic reticulum

**TABLE 2:** Information of the other ACS genes.

Gene	CDS (bp)	Exon	Intron	Amino acid (aa)	MW (Da)	pI	Localization
*ChAACS*	2019	18	17	673	74,924.77	6.14	cytoplasm
*ChACSF2*	1848	16	15	616	68,226.71	8.13	mitochondrion
*ChACSF3*	1761	10	9	587	65,045.76	6.75	mitochondrion
*ChAASDH*	3,321	15	14	1,107	123,626.02	6.24	plasma membrane

Subcellular localizations of *ChACSs* were predicted using wolfpsort software. The results revealed that the *ChACSMs*, *ChACSF2*, *ChACSF3*, *ChACSBG1*, *ChACSL4*, *ChACSS1*, and *ChACSS3* genes were located in the mitochondrion, the *ChAACS*, *ChACSL1*, *ChACSL3*, and *ChACSS2* genes in the cytoplasm, the *ChACSL5*, *ChACSL6*, *ChSLC27A2*, and *ChSLC27A6* genes in endoplasmic reticulum, the *ChAASDH*, *ChACSBG2*, *ChSLC27A3*, and *ChSLC27A5* genes in plasma membrance and others in peroxisome. The lengths, molecular weight and theoretical isoelectric point of ACS proteins exhibited substantial variation. The lengths of 26 ACS proteins ranged from 578 to 1,107 amino acids, the MW varied from 64,636.62 to 123,626.02 Da and the pI value changed from 6.14 to 8.92 (Tables 1, 2).

### Phylogenetic analysis and multiple alignments

**FIGURE 1:**
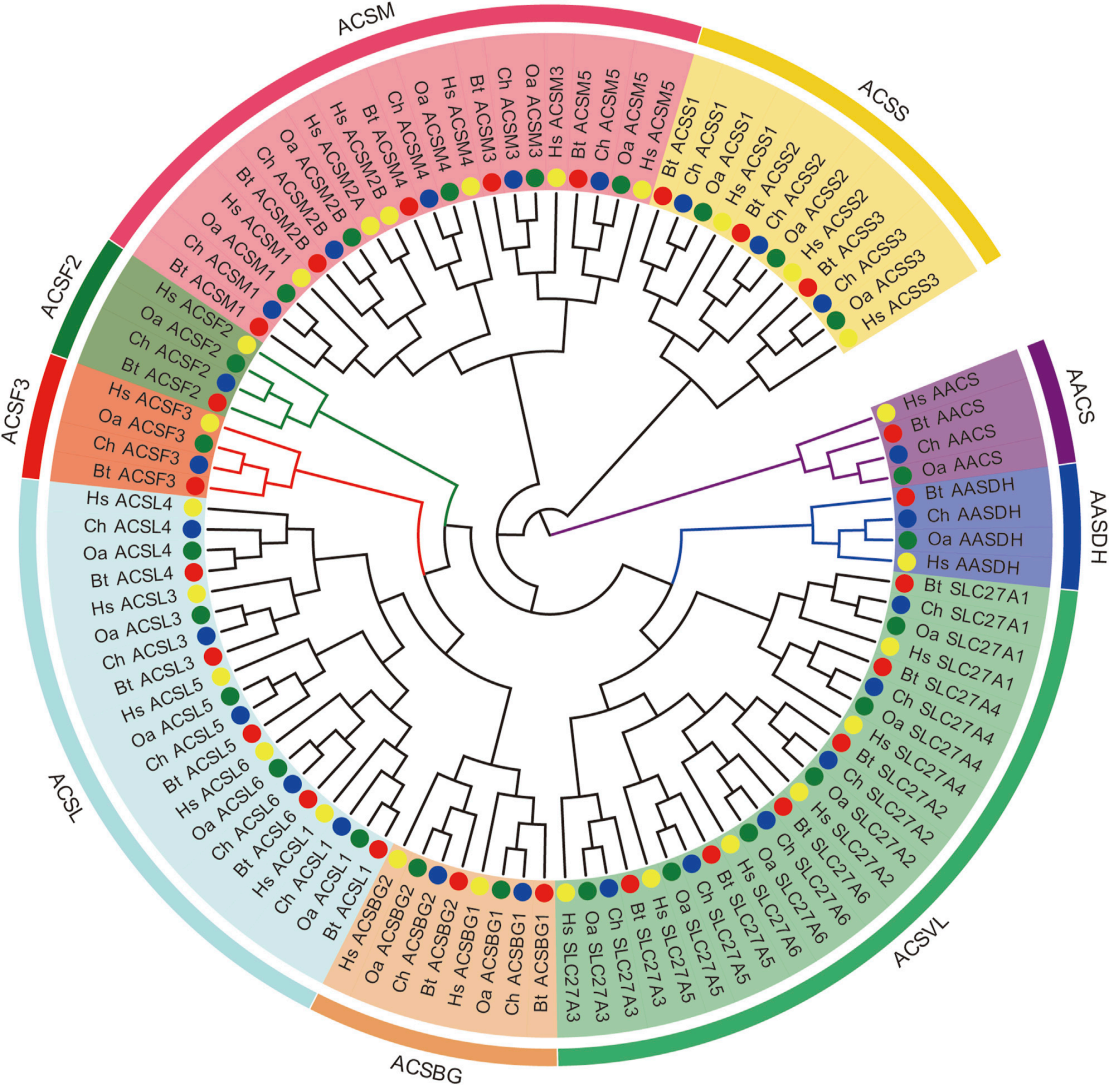
Phylogenetic tree of ACS proteins from *Capra hircus*, *Homo sapiens*, *Ovis aries*, and *Bos taurus*. Legends: The tree was generated using the IQ-TREE with an ultrafast bootstrap value of 1,000. Each ACS subfamiliy is marked by a different colour. Note: Ch, *Capra hircus*; *HS, Homo sapiens*; Oa, *Ovis aries*; Bt, *Bos taurus*.

In order to investigate the evolutionary relationships of the ACS proteins among human, goat, sheep, and cow, a phylogenetic tree was constructed based on their full-length protein sequences, using IQ-TREE software. According to the topological structure of the phylogenetic tree, all the ACS proteins are clustered into nine distinct clades, including the ACSS, ACSM, ACSL, ACSVL, and ACSBG families (Figure 1). The other four clades (ACSS, ACSF1, ACSF2, and AASDH) were not classified in the common evolutionary branch, and belonged to the greater ACS family (Watkins et al., 2007). Hereafter, we characterized these four genes using the ACSF group for facilitating description. Three species (goat, sheep, and cow) had the same amount of ACS proteins and the same gene name, except that the *ACSM2A* protein in the ACSM group. The ACS proteins of goat and sheep showed closer evolutionary relationships among three species. The ACSVL group was the largest branch of the ChACS phylogeny and contained six ChACS proteins.

**FIGURE 2:**
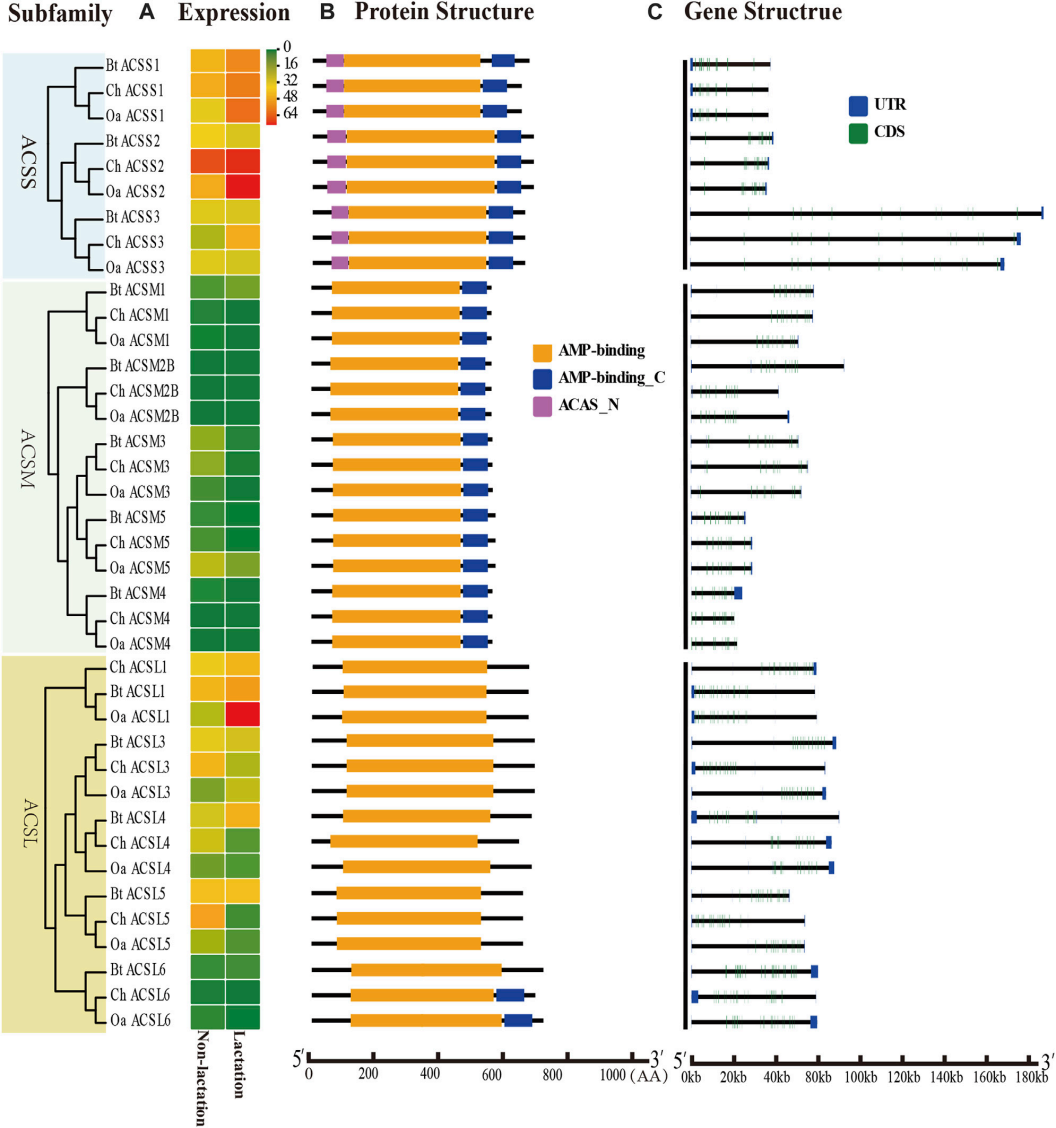
Expression profiles, gene structure and protein structure of ACS genes from the ACSS, ACSM, and ACSL subfamilies. Legends: **(A)** Heatmap showing the expression profiles of ACS genes in the non-lactation and lactation period of three species (dairy goat, sheep, and cow). Color gradient from green-to-red indicates expression values change from low to high. **(B)** Structures of ACS proteins with the AMP-binding domain represented by orange boxes, the AMP_C domain in red and ACAS_N domain in purple boxes. **(C)** Structure of ACS genes with exons in green, UTR regions in blue, and solid lines between the colored boxes representing introns.

**FIGURE 3:**
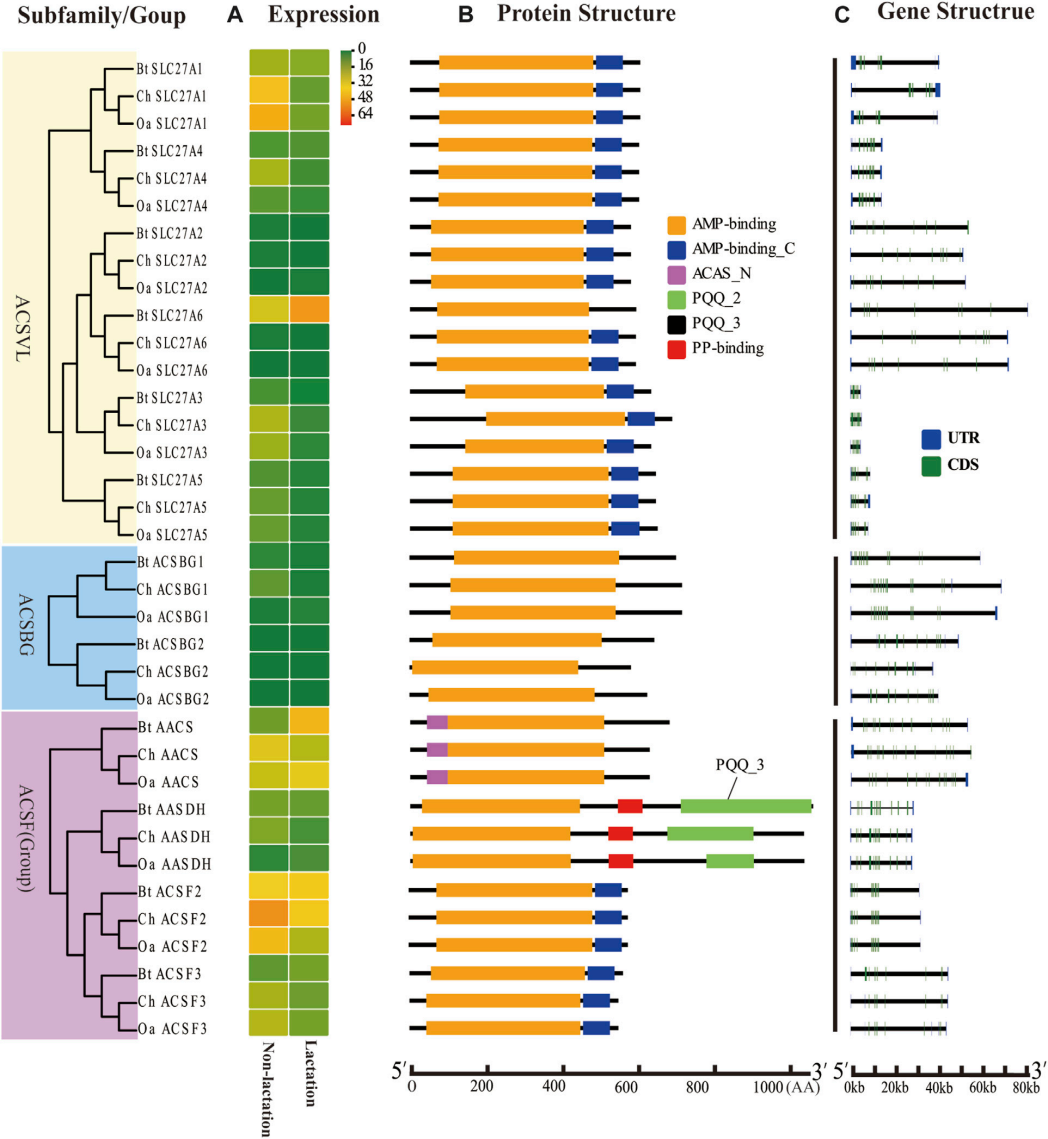
Expression profiles, gene structure, and protein structure of ACS genes from the ACSVL and ACSBG subfamilies and ACSF group. Legends: **(A)** Heatmap showing the expression profiles of ACS genes in the non-lactation and lactation period of three species (dairy goat, sheep, and cow). Color gradient from green-to-red indicates expression values change from low to high. **(B)** Structures of ACS proteins with the AMP-binding domain represented by orange boxes, the AMP_C domain in red, ACAS_N domain in purple, PQQ_2 domain in green, PQQ_3 domain in black and PP-binding domain in red. **(C)** Structure of ACS genes with exons in green, UTR regions in blue, and solid lines between the colored boxes representing introns.

To further characterize the ACS genes in goat, the protein sequences were aligned using the MAFFT-7.429. The conserved sequences of goat, sheep and cow ACS proteins were predicted by Pfam, and all proteins were found to contain AMP-binding domains (Figures 2B, 3B). In addition, the distribution of other protein domains was generally group specific. For instance, the acetyl-coenzyme A synthetase N-terminus (ACAS_N) domain was shared by all proteins in AACS subfamily. The AMP-binding enzyme C-terminal (AMP-binding_C) domain appeared in ACSS, ACSM, and ACSVL subfamily. Other domains, including phosphopantetheine attachment site (PP-binding), PQQ_2 and PQQ_3, were exclusive to AASDH proteins. Notably, *ChSLC27A6* and *ChACSL6* contained the AMP-binding_C domain, while *BtSLC27A6* and *BtACSL6* did not, indicating the function of *SLC27A6* and *ACSL6* genes might have changed in goat and cow (Figure 3B).

### acyl-coenzyme A synthetase gene structure, chromosomal location, and conserved motif analysis

The structural diversity of exon-introns is considered to play an important role in genetic evolution. Therefore, we performed exon–intron structure analysis to explore the structural evolution mechanism of the ACS genes using the GSDS tool. The result showed that the number of introns in the ChACS genes contained from 9 to 21 introns (Tables 1, 2), which was essentially consistent with what was found for the BtACS (Figures 2C, 3C). The exon-intron structure data also supported the phylogenetic tree topological structure. For example, the ACSVL subfamily contained 9–12 introns, and the ACSM subfamily had 12–14 introns (Table 1). The majority of ChACS genes had both 5′-and 3′-untranslated regions (UTRs), the *ChSLC27A3* contained a 5′ -UTR only, and the *ChACM4* contained no UTR region (Figures 2C, 3C).

**FIGURE 4:**
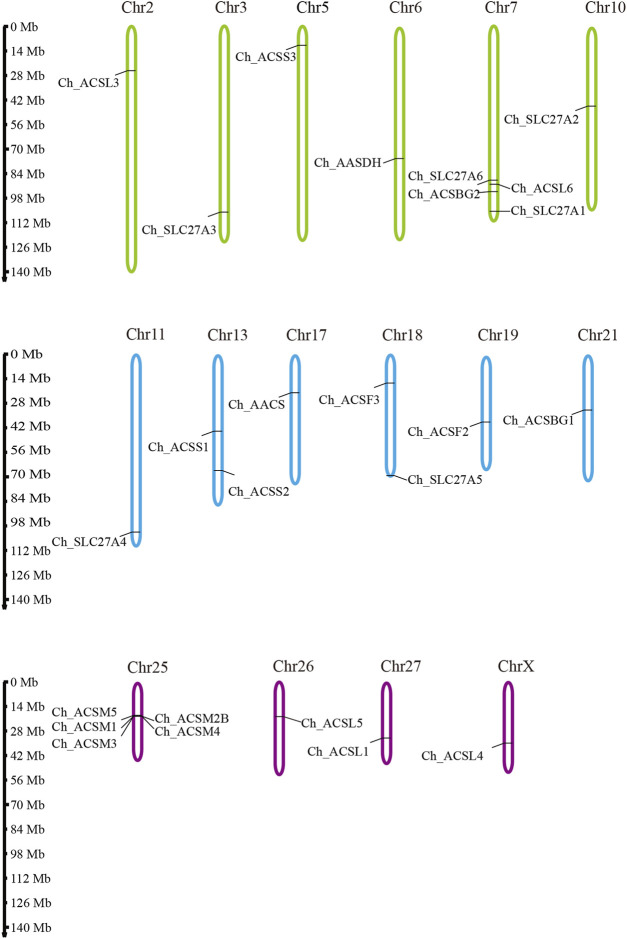
The chromosomal distribution of ChACS genes. Legends: The chromosomal position of each ACS gene was mapped to the goat genome. The chromosome number is indicated at the top of each chromosome.

To investigate the distribution of ChACS genes in the goat genome, the ChACS genes were mapped to individual chromosomes. The 25 ChACSs were distributed on 16 chromosomes, with five *ChACSs* on chromosome 25, four *ChACSs* on chromosome 7, two genes on chromosomes 25 and 28, and one *ChACS* on each of chromosomes 2, 3, 5, 6, 10, 11, 17, 19, 21, 26, 27, and X (Figure 4).

**FIGURE 5:**
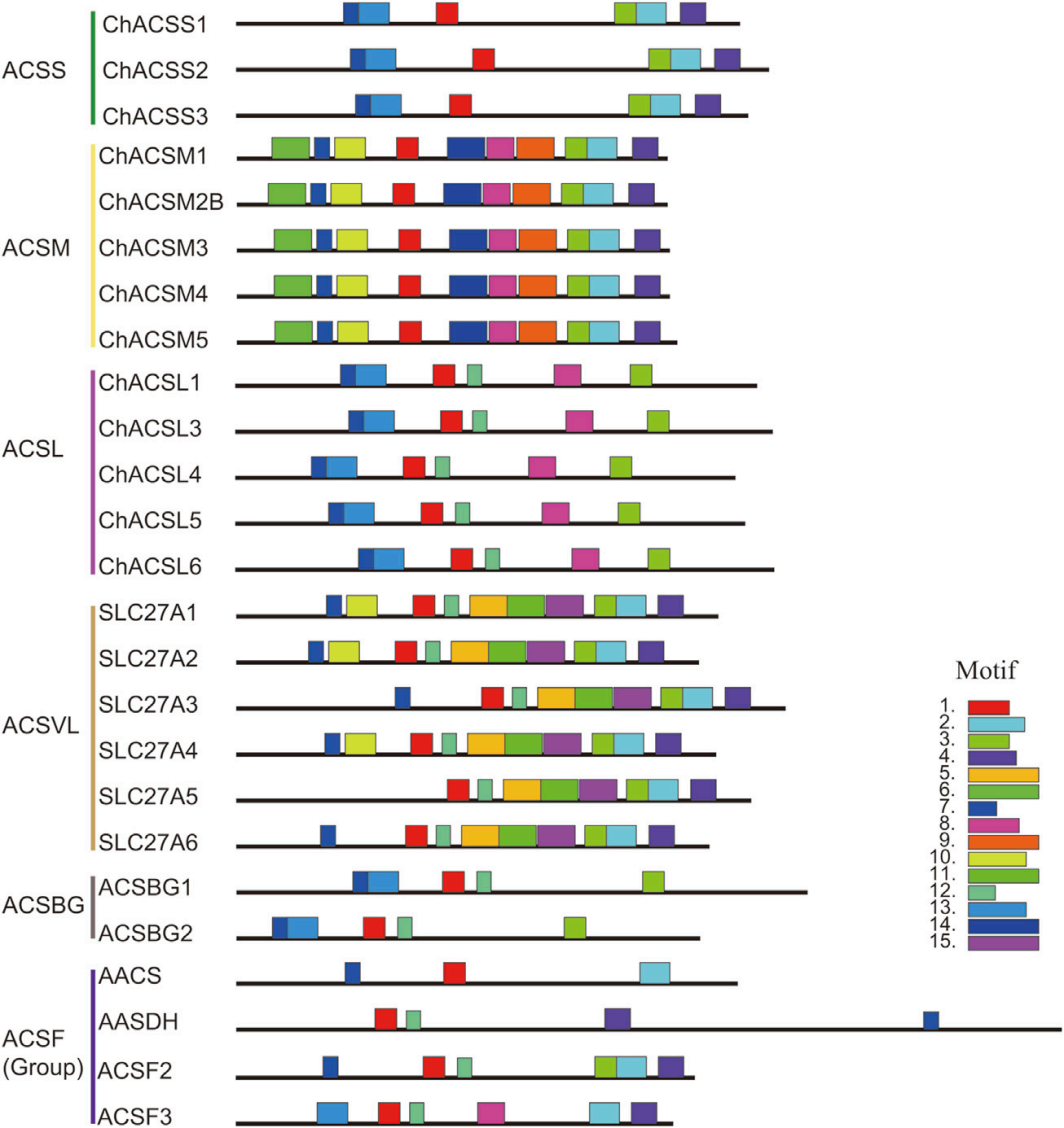
Motif distribution in ACS genes from goat. Legends: Motifs were predicted using the MEME web server (https://www.swissmodel.expasy.org/). The motifs are represented by different colors. The length of each box in the figure does not represent the actual motif size.

**FIGURE 6:**
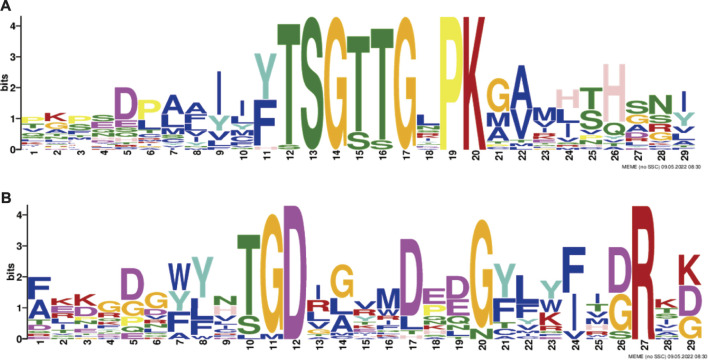
The conserved **(A)** motif 1 and **(B)** motif 3. Legends: These logos are graphic representations of amino acid sequences obtained from multiple sequence alignments. The motif 1 and motif 3, both of which were all components of the AMP-binding domain and played vital roles in substrate binding and/or catalysis (Watkins et al., 2007). The larger the fonts, the more conserved the motifs.

In this study, the MEME tool was used to identify conserved motifs. A total of 15 different conserved motifs were predicted in the ChACS proteins (Figure 5). All of the 25 ACS proteins contained Motif 1, *ChACSF3* did not contain Motif 7, while three proteins (*ChAACS*, *ChAACDH*, and *ChACSF3*) did not contain Motif 3. The closest ACS proteins in the phylogenetic tree had similar motifs. For example, each ACSM group member contained 10 motifs with similar motif composition pattern, which was very distinct from that of proteins in the other groups. Notably, more than 22 ACS proteins contained motif 1 and motif 3 (Figure 6), which were components of the AMP-binding domain and played vital roles in substrate binding and/or catalysis (Watkins et al., 2007).

### Expression analysis of acyl-coenzyme A synthetase genes

**FIGURE 7:**
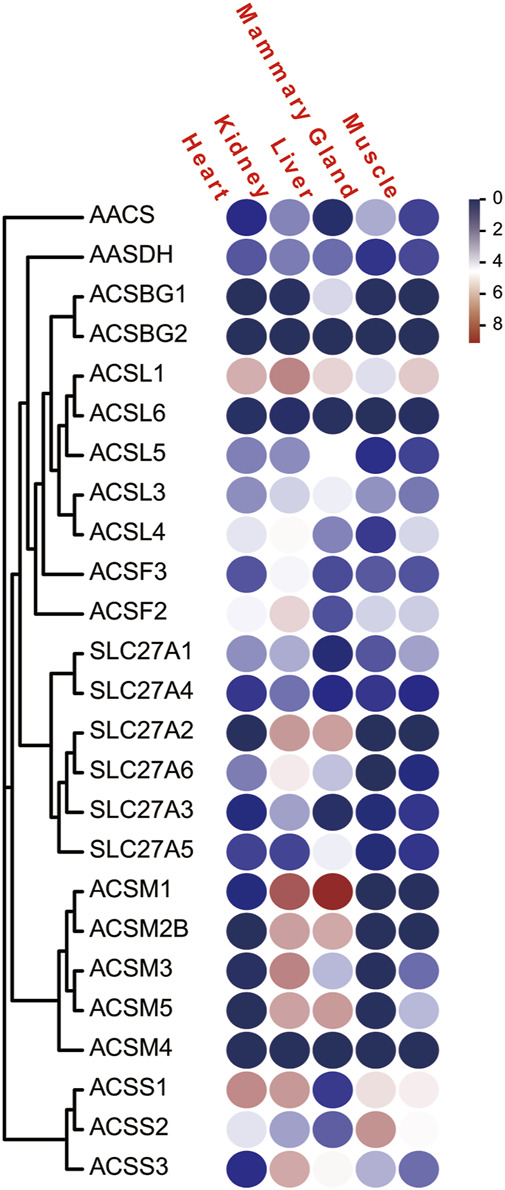
The FPKM of the of ACS genes of the dairy goat in five different tissues. Legends: The heat map was created with the log-transformed values of the FPKM values of ACS genes. Color gradient from navy-white-red indicates expression values change from low to high.

To analyze the expression profiles of ACS genes in distinct tissues of dairy goat, we investigated the FPKM values of the ACS genes in heart, kidney, liver, mammary gland, and muscle tissues. The majority of the 25 ACS genes were expressed in the test tissues, and the expression of the *ACSBG2* was not detected in multi tissues. Only *ACSL1* was highly expressed in all tissues. In general, the expression profiles analysis suggested that the expression of ACS genes of the dairy goat was related to distinct tissues and the expression patterns were also distinct among each ACS subfamily (Figure 7; Supplementary Sheet S3). For example, the members of the ACSM subfamily, except for *ACSM4*, exhibited higher expression level in kidney and liver than in other tissues. In heart, *ACSS1* was most highly expressed, and the ACS genes, including *ACSL1*, *ACSL4*, *ACSF2*, *ACSS1*, and *ACSS2*, were highly expressed. Most of ACS genes were highly expressed in kidney with *ACSM1* expression was prominent. Similarly, *ACSM1* was also highly expressed in liver. The majority of ACS genes exhibited lower gene expression in mammary gland and muscle tissues. The highest expression was observed in mammary gland and muscle for *ACSS2* and *ACSL1*, respectively. These finding suggested ACS genes had different functions in distinct tissues.

To explore the ACS genes expression profiles in mammary glands among dairy goat, sheep and cow, we compared their transcript abundance in the non-lactation and lactation period (Figures 2A, 3A). The expression pattern indicated that the expression levels of ACS genes was associated with each ACS protein group. For instance, the proteins of ACSM and ACSBG groups showed lower expression in three species, and members of ACSS and ACSL groups exhibited high expressions, except for *ACSL6*. Some ACS genes showed identical expression trend in dairy goat and sheep, while exhibited distinct expression patterns in cow (Supplementary Sheet S4). For example, the expression of *ChACSS2* and *OaACSS2* was the most highly expressed relative to other members in lactation stage, while *BtACSS2* was not preferentially expressed. In addition, the *ACSS2* exhibited higher transcript abundance level in lactation stage than in non-lactation stage in dairy goats and sheep, while the opposite was observed in cow. Whereas, the transcript abundance of *ACSL4* and *ACSF3* was significantly lower in lactation stage than in non-lactation stage in dairy goat and sheep, which showed opposite result in cow. Some genes such as *ACSM3*, *ACSM5*, and *ACSS1* exhibited the same expression trend in dairy goat and cow, which were lower expression level in non-lactation period than in lactation period. These differences in expression profiles of goat and cow suggested that the ASC genes might have an effect on composition of fatty acids in goat milk.

## Discussion

Previous studies have shown that fatty acids are activated to fatty acyl-CoAs by ACSs before involving in both anabolic and catabolic processes. Processes such as the synthesis of acylated protein and comple lipids, fatty acid extension or unsaturation, and fatty acid oxidation all require activated fatty acid substrates (Steinberg et al., 2000). This study found that a total of 25 full-length ACSs (without *ACSM2A*), representing five subfamilies (ACSS, ACSM, ACSL, ACSVL, ACSBG) and one group (ACSF) in the *Capra hircus* reference genome. In addition, we performed analyses of their phylogeny relationships, gene structures, conserved domains and motifs, expression profiling in multi tissues, and expressed difference in the non-lactation and lactation mammary glands of three species (goat, sheep, and cow).

There are 26 members of the ACS gene family reported in mammals (Watkins et al., 2007). Surprisingly, *ACSM2A* is missing in goat, sheep and cow genomes in the present study. The biological function of *ACSM2A* has rarely been reported in the current literature (Rencia and Erasmus, 2016; van der sluis et al., 2018). Several reports have shown that the *ACSM2A* and *ACSM2B* are nearly identical, with nucleotide homology of 98.8% and an amino acid identity of 97.1%. However, evidence also suggests that they are distinct genes (Rencia and Erasmus, 2016). Our findings showed the *ChACSM2B* was homologous to the *HsACSM2A* and *HsACSM2B* (Watanabe et al., 2020), with the amino acid identity of 80.6% and 80.4% respectively. Thus, *ChACSM2B* is highly conserved in the evolutionary process and might have similar function to *HsACSM2A*. Phylogenetic analysis exhibited that each evolutionary branch of the ACS family contained goat, sheep, cow and human proteins, suggesting that possible functions may be conserved among species (Liu et al., 2020). The four members of ACSF group, including *AACS*, *ACSF2*, *ACSF3*, and *AASDH*, clustered into four distinct clades, which was in consistent with the previous studies (Watkins et al., 2007). All the coding sequence of *ACSs* are disrupted by 9–21 introns, and the intron positions of goat and cow are distinct, suggesting that intron insertion may be result from independent events (Karan et al., 2003). All of ACS members contain related AMP-binding domains and FA binding motifs (Watkins et al., 2007). In this study, we found all ACS proteins shared a similar AMP-binding domain, which also illustrated that the AMP-binding functional domain was a conserved sequence and directly participated in the catalytic reaction (Wang et al., 2022). In addition to AMP-binding domain, the distribution of other protein domains was generally group specific. The conserved motif analysis showed that ACSs motifs also shared group specific, and the closest ACS proteins in the phylogenetic tree had similar motifs. These findings suggest that a wide range and diversity of ACSs might be result from suffering selective pressures to adapt to the metabolism of numerous and complex fatty acids in the evolutionary process (Watkins et al., 2007).

Each ACS gene plays a unique role, channeling its CoA derivatives to a specific metabolic pathway (Rencia and Erasmus, 2016). Fatty acyl-CoA molecules are the important regulatory molecules and metabolic intermediates (He et al., 2022), which have a variety of functions in the metabolism. Acyl-CoAs are oxidized to provide cellular energy, and are instrumental in the synthesis of acylated protein, and complex lipids such as triacylglycerols and phospholipids (Wang et al., 2022). Although *ACSL1* is highly expressed in heart, kidney, liver, mammary gland, and skeletal muscle, it has been shown to have different functions in different tissues because of the dual location on both the mitochondria and the endoplasmic reticulum (Grevengoed et al., 2014). The ACSM proteins are considered as liver mitochondrial enzymes (Rencia and Erasmus, 2016), which were also confirmed in this study. We also observed that the ACSM proteins were highly expressed in kidney, indicating that the ACSM family members might also play vital roles in kidney fatty acids metabolism. Several of the ACSVL family members located in the internal cellular membranes may have separate transport and activation functions, while ACSVLs not located on the plasma membrane are thought to enhance cellular uptake of fatty acids (Grevengoed et al., 2014). Multiple ACS proteins were expressed simultaneously in the same tissue, suggesting that these genes may coordinate together to perform a similar function. Several genes in the same subfamily exhibited distinct expression pattern from other members, such as *ACSL1* in ACSL subfamily and *SLC27A6* in ACSVL subfamily, suggesting that they may be involved in different biological functions.

vBCFAs are the cause of the goaty flavour of goat milk (Teng et al., 2018) and are more likely to be synthesized in goat tissues than in rumen microbes (Berthelot et al., 2001). It is believed that methylmalonate formed by carboxylation of propionic acid is an essential substance for the synthesis of vBCFAs (Priolo et al., 2001). Thus, *ACSs* might be involved in the synthesis or regulation of vBCFAs. By investigating the differences of expressed profiles in non-lactation and lactation mammary glands between goat and cow, it was possible to explore which ACS genes participate in formation of the goaty flavour of goat milk. In this study, the analysis of expression profiles suggests that *ACSF3* showed identical expression trend in dairy goat and sheep, while exhibited distinct expression patterns in cow. *ACSF3* is able to activate malonic acid to malonyl CoA (Chen et al., 2011), and also catalyzes methylmalonic acid to methylmalonyl-CoA (Monteuuis et al., 2017). It was reasonable to speculate that *ACSF3* provided the substrate for the synthesis of vBCFAs. ACS catalyzes propionate to propionyl-CoA which is a primer for the synthesis of 4-methyloctanoic acid (4-Et-8:0), and butyryl-CoA is the primer for the synthesis of 4-ethyloctanoic acid (4-Et-8:0). In this study, ACSS subfamily members were highly expressed, and the *ChACSS2* was the gene with the highest expression among all *ChACSs*, but *BtACSS2* was not preferentially expressed in cow. ACSSs typically activate short-chain fatty acids like acetate, propionate, or butyrate, involving in energy metabolism (Watkins et al., 2007). Our subcellular localization results have also proven that *ACSS1* and *ACSS3* were the mitochondrial proteins (Moffett et al., 2020; He et al., 2022), and *ACSS2* was localized in cytoplasm and nucleus (Li et al., 2017). As the fatty acids are activated in cytoplasm, our results suggest that *ACSS2* might participate in the synthesis of vBCFAs.

Goat milk contains high amounts of short-chain and medium-chain fatty acids (Luna et al., 2008). The oxidation of fatty acids is initiated in the cytoplasm by the formation of acyl-CoA by ACSs that are located in the endoplasmic reticulum and mitochondrial outer membrane (Watkins and Ellis, 2012). ACSMs typically activate medium-chain fatty acids, while ACSLs have a preference for long-chain fatty acids (Rencia and Erasmus, 2016). In this study, ACSMs were expressed at relatively low levels in lactation stage. We suggest that low expression of ACSMs is a possible reason for the high concentration of free medium-chain fatty acids in goat milk. We also observed that the genes including *ChACSL4*, *ChACSL5*, *OaACSL4*, and *OaACSL5* exhibited a lower transcript abundance in lactation stage than in non-lactation stage, but *BtACSL4* and *BtACSL5* showed opposite trend. Thus, we propose that *ACSL4* and *ACSL5* may have an effect on the long-chain fatty acid content between goat milk and cow’s milk.

ACSVL subfamily members are integral transmembrane proteins, which play a vital role in the absorption of long-chain fatty acids into cells (Gallardo et al., 2013). *ACSF2* is a mitochondrial matrix enzyme involved in the tricarboxylic acid cycle and fatty acid synthesis (Yang et al., 2019). *AASDH* is a protein of unknown function and homologous to bacterial non-ribosomal peptide synthetase (Drozak et al., 2014). Previous studies and the current research suggest these genes may not related to the metabolic process of vBCFAs.

## Conclusion

A total of 25 ACS genes were characterized in goats and subdivided into five subfamilies. The ACS proteins all had the conserved the AMP-binding domain and motif1. The phylogenetic relationships of *ACSs* were also supported by gene structures, motifs and protein domain. The majority of the ACS genes were expressed in the multi tissues, with similar or different expression levels. These findings provide reference information to further understand the classification and putative functions of ACS genes in goats. Two genes, *ACSS2* and *ACSF3*, may take part in the synthesis of vBCFAs. This study also provides genomic and expression information for *ACSs* in goat, and the findings may be useful for further research on the formation mechanisms of the goaty flavour in goat milk.

## Data Availability

The sequences information analyzed in this study are available in the GenBank (https://www.ncbi.nlm.nih.gov/), and the Ensemble database (http://ftp.ensembl.org/pub/release-104/). The RNA-seq data was from the NCBI’s SRA database (Accession: PRJNA309284, PRJNA309345, PRJNA339650, PRJNA637690, and PRJNA482783).

## Abbreviations

AACS, Acetoacetyl-CoA synthetase; AASDH, 2-Aminoadipic 6-semialdehyde dehydrogenase; ACAS_N, Acetyl-coenzyme A synthetase N-terminus; ACSs, Acyl-coenzyme A synthetase genes; ACSBG, Bubblegum acyl-coenzyme A synthetase; ACSF2, Acyl-CoA synthetase family member 2; ACSF3, Acyl-CoA synthetase family member 3; ACSL, Long chain acyl-coenzyme A synthase; ACSM Medium-chain acyl-coenzyme A synthetase; ACSS, Short-chain acyl-coenzyme A synthetase; ACSVL, Very long chain acyl-coenzyme A synthase; AMP-binding_C, AMP-binding enzyme C-terminal; Bt, *Bos taurus*; CDS, Coding sequence; Ch, *Capra hircus*; DHA, Omega-3 docosahexaenoic acid; ER, Endoplasmic reticulum; FAs, Fatty acids; HMM, Hidden Markov Model; Hs, *Homo sapiens*; MEME, Multiple expectation maximization for motif elicitation; MW, Molecular weight; NCBI, National Center for Biotechnology Information; Oa, *Ovis aries*; pI, Theoretical isoelectric point; PP-binding, phosphopantetheine attachment site; RPKM, Reads per kilobase per million mapped reads; UTRs, untranslated regions; vBCFAs, 4-alkyl-branched-chain fatty acids.
